# Genes Affecting Cotton Fiber Length: A Systematic Review and Meta-Analysis

**DOI:** 10.3390/plants14081203

**Published:** 2025-04-12

**Authors:** Jiao Jiao, Shihao Chang, Fei Wang, Jiaxin Yang, Asigul Ismayil, Peng Wu, Lei Wang, Hongbin Li

**Affiliations:** Ministry of Education Key Laboratory of Xinjiang Phytomedicine Resource Utilization, Xinjiang Production and Construction Corps Key Laboratory of Oasis Town and Mountain-Basin System Ecology, College of Life Sciences, Shihezi University, Shihezi 832000, China; j2023j@126.com (J.J.); shihao2553@126.com (S.C.); feiw@shzu.edu.cn (F.W.); yangjiaxinyangle@126.com (J.Y.); asgli12@163.com (A.I.)

**Keywords:** CRISPR, VIGS, RNAi, meta-analysis, cotton fibers

## Abstract

Cotton fiber length is an important measurement for application in the textile industry, and researchers are seeking to cultivate cotton plants with longer fibers. In this study, cotton fiber genes were systematically reviewed through meta-analysis in terms of extending and shortening fiber and the use of different research technologies for the first time. PubMed, Web of Science, China National Knowledge Infrastructure (CNKI), and Baidu Xueshu databases were included as literature retrieval sources. A total of 21,467 articles were retrieved, and 45 articles were used in the final analysis. Data analysis was performed using RevMan 5.4 software. To shorten cotton fiber length, clustered regularly interspaced short palindromic repeats (CRISPR)-Cas9 technology was superior to virus-induced gene silencing (VIGS) technology and RNA interference (RNAi) technology [*p* = 0.002, MD = −1.05, 95% CI (−1.73, −0.37), Chi^2^ = 39.89]. To increase cotton fiber length, CRISPR-Cas9 technology had a similar effect as VIGS technology [*p* = 0.12, MD = −0.59, 95% CI (−1.33, −0.15), Chi^2^ = 0.17]. When some genes (*GhLAC15*, *GhALDH7B4*, *GhMDHAR1A*/*GhDHAR2A*, *STTM-miR396b*, *GhMYB44*, *GhFP2*, *GhMYB7*, *GhKNL1*, *GhTCP4*, *GhHDA5*, *GhGalT1*, *GhKNOX6*, *GhXB38D*, and *GhBZR3*) were damaged, cotton fiber length increased. Furthermore, we found that after gene interference, the fiber-shortening genes occurred more frequently than the fiber-elongating genes. Synergistic research on these genes may better promote cotton fiber elongation.

## 1. Introduction

Studying the genotype of cotton, one of the most important agricultural crops, is of great significance [1]. Cotton fiber length serves as one of the key indicators for assessing the cotton quality [2]. There exists a positive correlation between cotton fiber length and yarn strength: longer fibers exhibit an increased interlocking length during spinning processes, thereby reducing fiber slippage probability. This mechanism requires overcoming greater frictional resistance during yarn fracture, significantly enhancing yarn strength. The reduced exposure of fiber ends in long-staple cotton spinning decreases yarn hairiness rate by 30–50%, improving fabric surface smoothness by over 30% [3]. Long-staple cotton fibers generally exhibit 15–20% higher tensile strength than regular cotton, enabling textile products to achieve 1.8 times the abrasion resistance cycles of conventional counterparts. This exceptional performance grants long-staple cotton irreplaceable status in premium yarn-dyed fabrics and home textile applications [4]. A nation’s production capacity in long-staple cotton directly dictates its competitive edge within the high-end textile industrial chain, making fiber length enhancement strategically significant for industrial upgrading and value chain advancement [5]. The regulatory role of genes in cotton fiber development is divided into two categories: elongation and shortening. However, the specific genes promoting fiber elongation versus those contributing to fiber shortening have not been systematically elucidated.

In recent years, clustered regularly interspaced short palindromic repeat (CRISPR) gene editing technology and virus-induced gene silencing (VIGS) have been increasingly applied in cotton fiber research. CRISPR-based editing has been developed and widely used based on the advantages of high efficiency and ease of use, enabling transgene-free, gene-edited crops [6]. CRISPR-Cas9 is an RNA-guided genome editing technology for genetic engineering that can be guided to specific locations in genomes using a short RNA [7], which commonly refers to edits caused by non-homologous end joining repair at nuclease cut sites [8,9,10,11]. CRISPR-Cas9 is more accurate, faster, and more cost-effective than other genome editing methods [12]. RNA interference (RNAi) is more popular than the anti-sense technology that was highlighted earlier due to its high efficiency, accuracy, and stability [13]. RNAi technology involves the regulation of specific gene sequences by small interfering RNA (siRNA) and has become a promising method for studying plant functional genomics. VIGS, a powerful tool, has been widely used in plant functional genomics [14]. When inoculated with the VIGS vector carrying the target gene, plants generate siRNA and further activate the RNA-induced silencing complex to degrade homologous endogenous transcripts, thus silencing the host gene. VIGS is fast and simple and silences genes in a transient manner [15,16,17,18].

Meta-analysis, a statistical method, provides the highest level of evidence and has been widely applied in evidence-based medicine. It integrates multiple independent original research data to draw comprehensive conclusions [19] and obtain more reliable and powerful evidence than individual research studies, and it has the advantages of improving statistical efficiency, reducing bias, providing more accurate estimates, and exploring heterogeneity [20].

**Figure 1:**
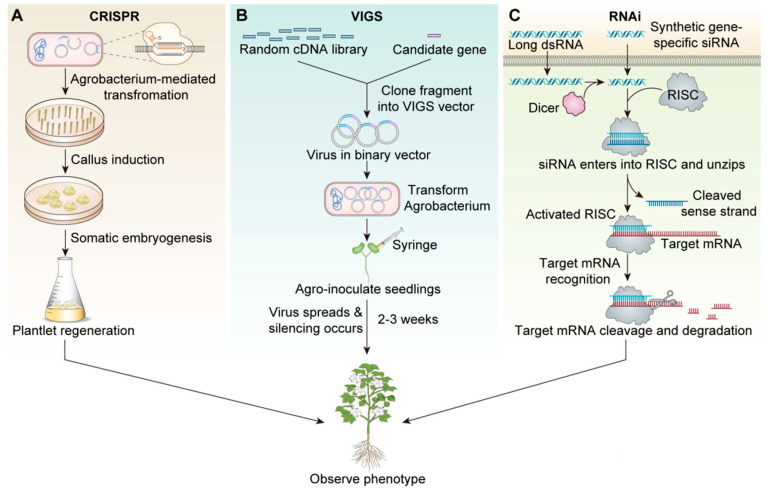
The basics of the technologies of CRISPR, VIGS, and RNAi technology in cotton fiber research. (**A**): CRISPR-Cas9 technology schematic. (**B**): VIGS Technical Schematic. (**C**): Schematic diagram of RNAi technology.

Gene knockout or knockdown technology introduces specific mutations in gene sequences that prevent the target gene from being expressed normally, or completely lose its function. Its significance lies in helping researchers understand the functions of specific genes in organisms, and in the agricultural field, by knocking out or knocking down certain genes, researchers can improve crop yield, disease resistance, or nutritional value. RNAi and CRISPR-Cas9 are the most popular and commonly used technologies in cotton fiber knockdown or gene knockout research. In cotton cultivation, RNAi-derived biopesticides have achieved commercial adoption, though RNAi’s historical prominence in gene function studies is being challenged by CRISPR-based systems. Mechanistically, RNAi primarily operates through cytoplasmic RNA interference, presenting limitations in targeting nuclear-localized transcripts—a challenge CRISPR readily addresses via its DNA-guided nuclease activity in the nucleus [21]. However, differences in application among CRISPR-Cas9, VIGS, and RNAi have not been well studied (Figure 1).

This study presents the first comprehensive investigation and systematic evaluation of both genetic and technological factors associated with fiber length development. For precise exploration, we performed categorical analysis of genes contributing to both fiber elongation and shortening. To ensure methodological rigor and data reliability, we conducted independent analyses of three commonly used technologies. Our findings provide researchers with an integrated and visually accessible reference. The most significant value of this research lies in its accurate and systematic assessment of genetic influences on cotton fiber length. Furthermore, the comparative analysis of technologies provides valuable guidance for selecting appropriate methodologies in commercial applications.

## 2. Results

**Figure 2:**
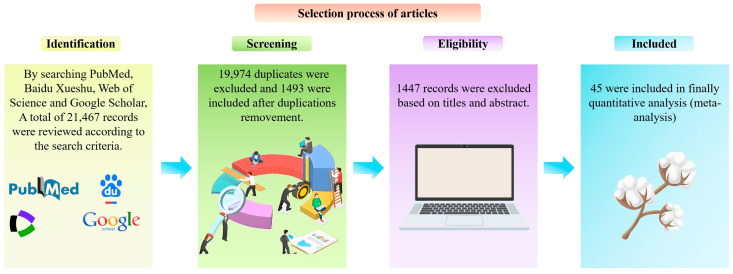
Flowchart of literature retrieval.

To investigate and systematic evaluate genes and technological factors associated with fiber length development, using PubMed, Web of Science, CNKI, and Baidu Xueshu databases, a comprehensive literature search was conducted for genes related to fiber development that have been reported (Figure 2). According to the literature inclusion and exclusion criteria, a total of final 45 articles were included for further meta-analysis, and the characteristics of each article were extracted, including the first author, publication time, technology used, and cotton fiber length (Figure 2).

The publication period of the included literature was 2008–2024. The data on cotton fiber were extracted using the terms “CRISPR-Cas9”, “VIGS”, and “RNAi”, and then included for meta-analysis (Table 1).

After different gene expressions were inhibited, the length of cotton fiber showed an increasing or decreasing trend. Systematic studies and analyses of these genes may better promote the growth of cotton fiber. Only one technology was used in most of articles, while both CRISPR and VIGS technologies were used at the same time in three papers. Few studies reported the role of the same gene, but *MYB* appeared three times, which may be related to the novelty of the published articles. The genes associated with fiber development was summarized in Table 1. The most studies are reported in 2023 and 2024.

**Figure 3:**
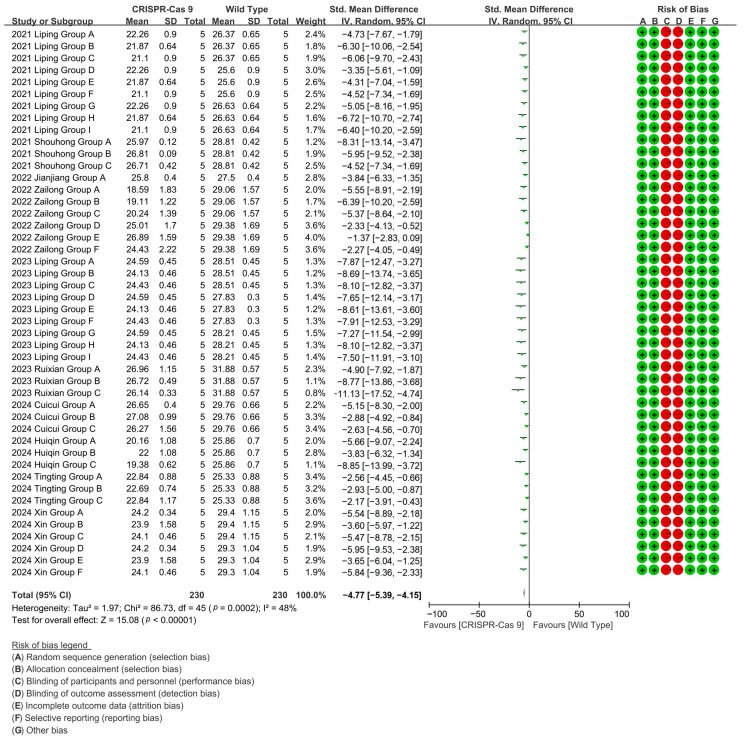
Forest plot of CRISPR-Cas9 for shortening the cotton fiber length.

CRISPR-Cas9 editing achieved 84% suppression of wild-type cotton fiber length in a 46-group meta-analysis (10 studies), mediating directional fiber shortening phenotypes [*p* < 0.00001, MD = −4.77, 95% CI (−5.39, −4.15), Chi^2^ = 86.73] (Figure 3).

**Figure 4:**
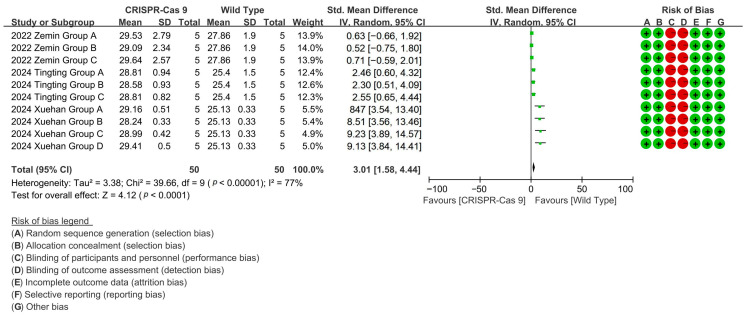
Forest plot of the effect of CRISPR-Cas9 on increasing the cotton fiber length.

To evaluate the effect of CRISPR-Cas9 editing on increasing the cotton fiber length, the analysis of three related studies demonstrated that CRISPR-Cas9 editing enhanced cotton fiber length by up to 111.5% through targeted gene modifications [*p* < 0.0001, MD = 3.01, 95% CI (1.58, 4.44), Chi^2^ = 39.66] (Figure 4).

**Figure 5:**
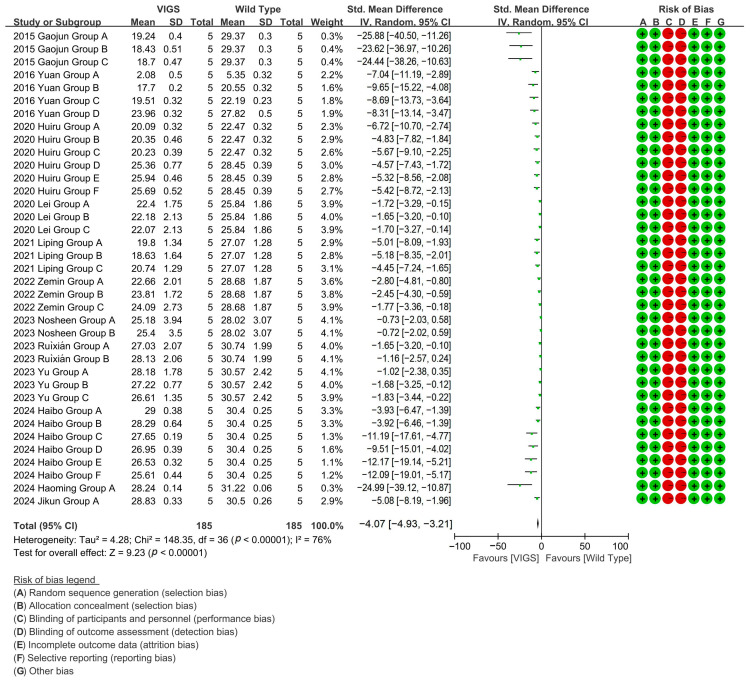
Forest plot of VIGS for shortening the cotton fiber length.

Because the gene silencing caused by VIGS is almost non-inherited, in most cases, it does not remain silenced in the next generation of plants, making it ideal for rapid preliminary functional analysis. It can be used to process multiple genes at the same time, and to perform large-scale gene function screening. In cotton research, VIGS can be used to quickly identify and validate gene functions related to fiber development, stress resistance, yield, and other traits, providing candidate genes for subsequent CRISPR editing [66]. The advantages of VIGS in high-throughput gene function screening make it ideal for the early stages of research, especially in cotton varieties that lack genetic transformation systems [67]. To evaluate the effect of VIGS technology on the cotton fiber length, meta-analysis of 12 studies (37 experimental groups) revealed that VIGS-mediated gene silencing reduced cotton fiber length by 85% versus wild-type (WT), specifically targeting fiber-growth genes with significant efficacy [*p* < 0.00001, MD = −3.97, 95% CI (−4.81,−3.14), Chi^2^ = 148.36] (Figure 5).

**Figure 6:**
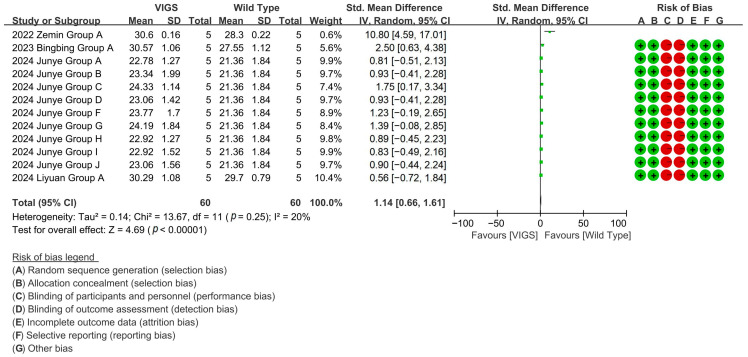
Forest plot of VIGS for increasing the cotton fiber length.

Through 12 experimental cohorts across four studies, VIGS-mediated silence of fiber shortening genes highlights a 108.65% increase in cotton fiber length compared to baseline controls [*p* < 0.00001, MD = 2.12, 95% CI (1.75, 2.49), Chi^2^ = 12.07] (Figure 6).

**Figure 7:**
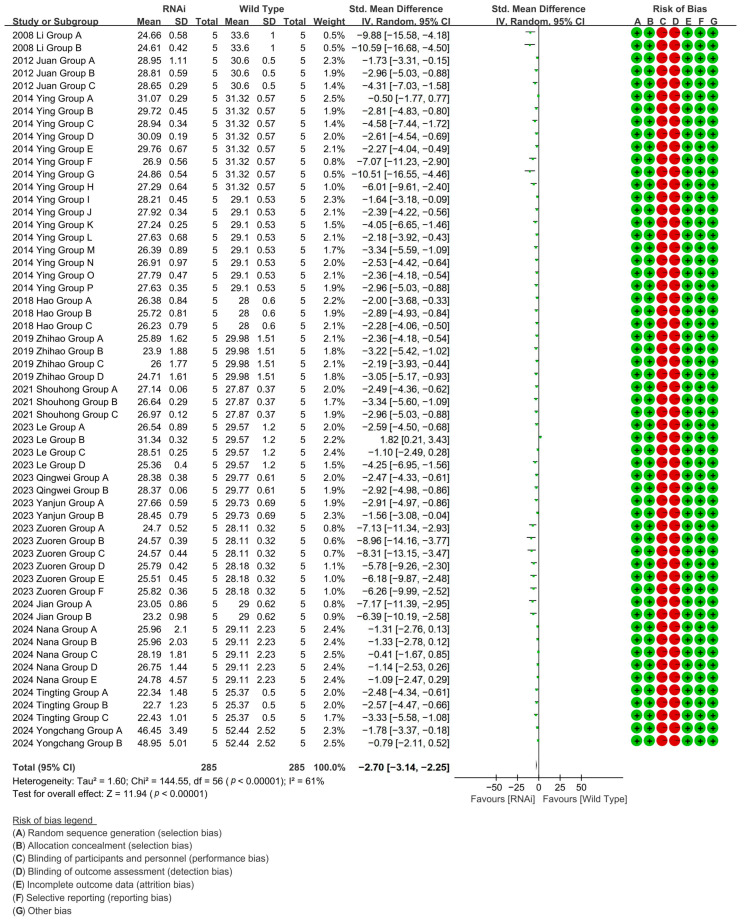
Forest plot of RNAi for shortening the cotton fiber length.

To investigate the effect of RNAi technology on the cotton fiber length, RNAi-mediated suppression in 57 experimental cohorts (14 studies) confirmed fiber-growth genes, reducing cotton fiber length by 90.66% versus WT controls [*p* < 0.00001, MD = −2.70, 95% CI (−3.14, −2.25), Chi^2^ = 144.55] (Figure 7).

**Figure 8:**
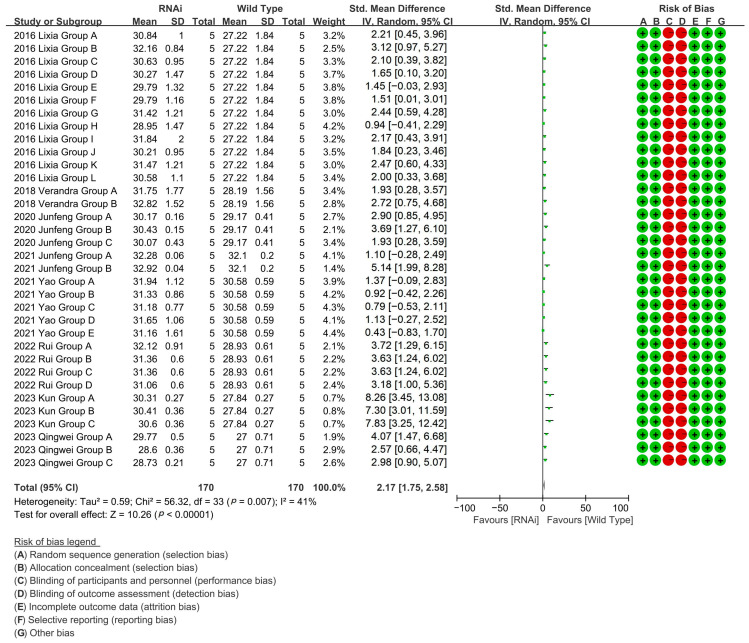
Forest plot of RNAi for increasing the cotton fiber length.

RNAi-targeted validation of fiber growth genes across 34 experimental groups (eight studies) enhanced cotton fiber length by 108.48% relative to controls [*p* < 0.00001, MD = 2.17, 95% CI (1.75, 2.58), Chi^2^ = 56.32] (Figure 8).

**Figure 9:**
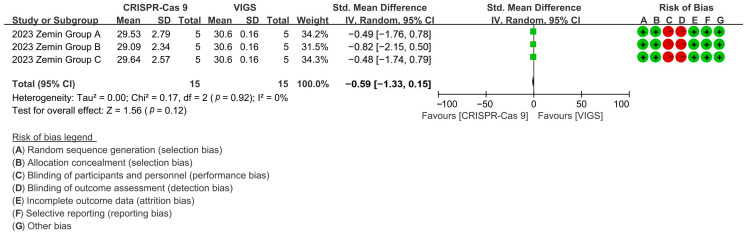
Forest plot of CRISPR-Cas9 and VIGS for increasing the cotton fiber length.

Comparative analysis revealed CRISPR-Cas9 and VIGS exhibited equivalent fiber growth modulation, with CRISPR editing reducing fiber length by 96.14% versus VIGS controls [*p* = 0.12, MD = −0.59, 95% CI (−1.33, −0.15), Chi^2^ = 0.17] (Figure 9).

**Figure 10:**
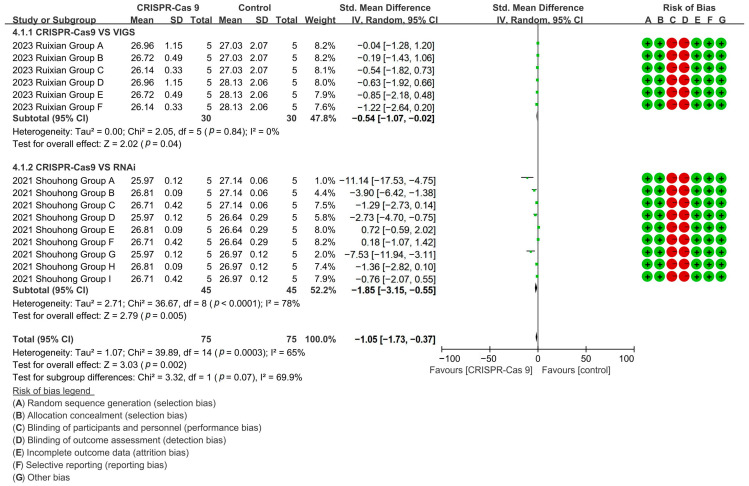
Forest plot of RNAi, CRISPR-Cas9, and VIGS for cotton fiber length shortening.

CRISPR-Cas9 technology demonstrated superior fiber shortening (96–98% reduction) versus both VIGS [*p* = 0.04, MD = −0.54, 95% CI (−1.07, −0.02), Chi^2^ = 2.05] and RNAi technology [*p* = 0.005, MD = −1.85, 95% CI (−3.15, −0.55), Chi^2^ = 36.67] (Figure 10).

## 3. Discussion

CRISPR, VIGS, and RNAi are three different gene knockout or knockdown technologies, and their applications in cotton fiber research have their own characteristics. CRISPR technology has performed well in cotton fiber research because of its high efficiency and accuracy. By precisely editing targeted genes, CRISPR technology has significantly increased the length and strength of cotton fibers, improving cotton yield and quality. In contrast, the VIGS technique is based on virus-mediated gene silencing, and involves interference with the expression of target genes to study the role of genes in cotton fiber development. However, VIGS technology has a complex effect on cotton fibers, sometimes resulting in a reduction in fiber length or quality, which may be related to the non-specific silencing effect of VIGS. However, this requires further study and optimization. RNAi technology uses double-stranded RNA molecules to specifically degrade target mRNA, thereby achieving gene silencing. It is widely used in cotton fiber research. By knocking down key genes, RNAi affects the growth and development process of cotton fibers, resulting in changes in fiber length, strength, and other traits. However, the effects of RNAi are likely to be mild compared with those of CRISPR, and the value of increase or decrease in cotton fibers is smaller.

The effects of VIGS technology and RNAi technology are not permanent, and it weakens with time. CRISPR gene editing technology can make the edited cotton fiber traits stably inherited. While CRISPR-Cas9 offers high precision and rapid editing capabilities, its technical complexity necessitates rigorous biosafety evaluations prior to agricultural deployment. VIGS, a transgene-free approach requiring no genetic transformation, faces limited scalability in crop production due to its non-heritable modifications. RNAi enables sequence-specific gene silencing; however, RNA-based biopesticides derived from this technology exhibit transient efficacy, demanding repeated applications for sustained pest control. Herbicide-resistant crops may increase pesticide residue and change the soil microbial communities. In terms of regulations, RNAi technology products need to undergo strict environmental and food safety assessment to ensure their safety and effectiveness. Insecticidal characteristics might harm non-target pollinators, and genes flowing to wild plants are at risk of changing pollination dynamics. In agricultural applications, VIGS can be used to study the resistance mechanisms of crops to pathogens, but its practical application is limited [68]. Through CRISPR technology, researchers can create cotton varieties with specific traits, which can accelerate the cultivation process of excellent varieties. However, the cost of CRISPR technology is high, and requires fine operation and strict quality control. Therefore, sufficient technical optimization and cost evaluation are needed before large-scale application.

The global CRISPR technology market is valued at USD 3.4 billion in 2023, and is expected to reach USD 7.5 billion by 2029, with a compound annual growth rate (CAGR) of 14.4%. The RNAi technology market is expected to reach USD 4.8 billion by 2029, with an average annual compound growth rate of 14.8%. RNAi and VIGS technologies offer cost-effective solutions for cotton improvement, yet their modified fiber traits lack heritability, making them suitable primarily for foundational research [69]. VIGS, widely used in gene function studies, exhibits transient efficacy limited to the cotyledon stage of cotton seedlings, rendering it impractical for agricultural production; its commercial value lies in rapid gene discovery [70]. RNA-based biopesticides derived from RNAi boast broad-spectrum pest targeting, low development costs, and environmental safety, but face critical challenges including pest resistance evolution and ecosystem risks from off-target effects [71]. Successful CRISPR deployment could reduce cultivation expenses while elevating cotton’s economic viability.

There is no consistent and clear regulatory policy for CRISPR-Cas9, RNAi, and VIGS in the world, and the potential technical limitation of CRISPR-Cas9 lies in off-target effect. RNAi and VIGS technologies may face the problems of poor specificity, low efficiency and unstable transmission.

For gene technology, the main challenge still lies in the complexity of management and supervision. There are significant differences in regulatory policies, labeling requirements and safety assessment of gene editing in different countries, which may become an obstacle to international trade and technology transfer. CRISPR-Cas9, RNAi, and VIGS are widely utilized genetic engineering tools in plant research. The discussion on the influence of gene editing technology on natural evolution may spread genes to ecosystems and disturb natural selection [72]. Among these, CRISPR-Cas9 and RNAi face stringent global regulatory oversight due to their proven agricultural applicability and biosafety considerations. CRISPR-Cas9, distinguished by its high editing efficiency and user-friendly design, holds transformative potential for crop improvement. However, its commercialization is complicated by region-specific regulatory frameworks and mandatory adherence to biosafety protocols and ethical guidelines [73,74].

It is important to acknowledge certain limitations and heterogeneity in this study. The sources of heterogeneity were as follows: Firstly, although the average length of fibers is mentioned in the literature, there are no reports on the number of fibers. In future cotton research, data on cotton fiber quantity should be increased. Secondly, the differences in the results may be due to the different processing methods for cotton. In this study, we have included three common existing technologies for meta-analysis as three technical subgroups, and for each subgroup, we analyzed the genes for fiber elongation and fiber shortening separately after silencing, so we have six subgroups in total. Although it may seem a bit excessive, this is also where our advantage lies. Thirdly, some data may have been ignored, for example, using different databases and keywords. Results with significant differences are readily published, which may also lead to data deviation [75]. The last, the biggest heterogeneity of this study may be that after the gene was edited, it may increase or shorten the fiber. Independent analysis and subgroup analysis were carried out on the growing and shortening studies. We used PRISMA to evaluate the whole paper. RevMan 5.4 software of the Cochrane collaboration network was used to evaluate the quality of the data included. It was found that the source of heterogeneity may be that no double-blind experiments have been reported. This is often neglected in plant research. It is easier for researchers to know which ones are the experimental group and which ones are the control group. It is suggested to conduct the double-blind experiments on transgenic plants ready for commercialization.

## 4. Materials and Methods

### 4.1. Data Sources and Retrieval Methods

A comprehensive search was independently conducted using PubMed, Web of Science, CNKI, and Baidu Xueshu databases before October 2024 using “cotton”, “fiber”, and “CRISPR-Cas9 or VIGS or RNAi” as search terms. After searching these databases, the documents to be included were extracted, sorted, and reviewed.

### 4.2. Literature Inclusion and Exclusion Criteria

The inclusion criteria were as follows: ① clear descriptions of experimental types and methods; ② the object of study was cotton fibers; and ③ clear data on cotton fiber in the study. The exclusion criteria were as follows: ① literature review without cotton fiber data; ② literature on other plants as research objects; ③ data presented without the standard deviation; and ④ literature on non-genetically modified plants.

### 4.3. Literature Screening and Data Extraction

After retrieval was completed, duplicate documents were removed using literature king software 6.3.1.2. We read the titles and abstracts, and deleted any documents that were inconsistent with the theme. The full text was read, and documents that failed to meet the presented criteria were removed. The mean and standard deviation of cotton fiber data were extracted from the remaining literature, and the data were reorganized in a table.

### 4.4. Data Analysis

Meta-analysis of continuous variables was performed using Review Manager 5.4 software. The random-effect model was selected, and a forest map was used in the meta-analysis to determine whether there was a significant difference among cotton fiber length. The mean difference (MD) and 95% confidence interval (CI) for the effect values were calculated. Due to the wide application of VIGS, it was analyzed independently of RNAi. The data on cotton fibers were grouped and analyzed using the search terms “VIGS”, “CRISPR-Cas9”, and “RNAi”. First, the fiber length data sources obtained from these technologies were compared with those of the wild type (WT). Then, data sources for CRISPR-Cas9, VIGS, and RNAi were compared with each other. We presumed that all measurements met industry standards; thus, we set the value of each group to 5 [76]. During these analyses, we classified the genes responsible for cotton fiber elongation and shortening because they had opposite effects.

## 5. Conclusions

Different knockout methods have different effects on cotton fibers. When selecting a knockout method, comprehensive consideration should be given to the specific research purpose and experimental conditions. To shorten the cotton fiber length, CRISPR-Cas9 technology was superior to VIGS and RNAi technology (*p* = 0.002). To increase the cotton fiber length, CRISPR-Cas9 technology had the same effect as VIGS technology (*p* = 0.12). Furthermore, we found that after gene interference, the fiber-shortening genes appeared more frequently than the fiber-elongating genes. Synergistic research on these genes may better promote cotton fiber elongation.

## Figures and Tables

**Table 1:** Information on the cotton fiber literature included in the article.

Number	Author	Year	Technologies	Gene	Cotton Fiber Length	References
1	Cuicui Wu	2024	CRISPR	*GhHDZ76*	Shortening	[22]
2	Jian Zhang	2024	RNAi	*GhLCBK1*	Shortening	[23]
3	Haibo Zhang	2024	VIGS	*GhPDCB9*	Shortening	[24]
4	Haoming Mao	2024	VIGS	*GhEB1C*	Shortening	[25]
5	Huiqin Wang	2024	CRISPR	*GhFAD3-4*	Shortening	[26]
6	Jikun Song	2024	VIGS	*STTM-miR477b*	Shortening	[27]
7	Junye Jiao	2024	VIGS	*GhLAC15*	Increasing	[28]
8	Liyuan Tang	2024	VIGS	*GhALDH7B4*	Increasing	[29]
9	Na-Na Wang	2024	RNAi	*GhHOX4*	Shortening	[30]
10	Tingting Jia	2024	RNAi	*GhBLH1*	Shortening	[31]
11	Tingting Jia	2024	CRISPR	*GhFAD7A-1*	Shortening	[31]
12	Tingting Jia	2024	CRISPR	*GhKNOX6*	Increasing	[31]
13	Xin Li	2024	CRISPR	*GhATL68b*	Shortening	[32]
14	Xuehan Tian	2024	CRISPR	*GhMDHAR1A/GhDHAR2A*	Increasing	[33]
15	Yongchang Liu	2024	RNAi	*GhZFP8*	Shortening	[34]
16	Bingbing Zhang	2023	VIGS	*STTM-miR396b*	Increasing	[35]
17	Kun Xing	2023	RNAi	*GhMYB44*	Increasing	[36]
18	Le Liu	2023	RNAi, VIGS	*GhBES1.4/GhCYP84A1/GhHMG1*	Shortening	[37]
19	Liping Zhu	2023	CRISPR	*GhEXPA3-1*	Shortening	[38]
20	Nosheen Kabir	2023	VIGS	*GhTBL7/GhTBL58*	Shortening	[39]
21	Qingwei Song	2023	RNAi	*GhXB38D*	Increasing	[40]
22	Qingwei Song	2023	RNAi	*GhMAP20L5i*	Shortening	[41]
23	Ruìxián Liú	2023	CRISPR, VIGS	*GhCesA4*	Shortening	[42]
24	Yanjun Guo	2023	RNAi	*GhGT47B*	Shortening	[43]
25	Yu Gu	2023	VIGS	*GhKRP6*	Shortening	[44]
26	Zuoren Yang	2023	RNAi	*GhBES1.4, GhKCS10_At*	Shortening	[45]
27	Jianjiang Ma	2022	CRISPR	*Ghmah1*	Shortening	[46]
28	Rui Lu	2022	RNAi	*GhFP2*	Increasing	[47]
29	Zailong Tian	2022	CRISPR	*Ghd27, Ghgrf4*	Shortening	[48]
30	Zemin Shi	2022	CRISPR, VIGS	*GhBZR3*	Increasing	[49]
31	Zemin Shi	2022	VIGS	*GhKCS13*	Shortening	[49]
32	Liping Zhu	2021	CRISPR	*GhARF18*	Shortening	[50]
33	Liping Zhu	2021	VIGS	*GhPIPLC2D*	Shortening	[50]
34	Junfeng Huang	2021	RNAi	*GhMYB7*	Increasing	[51]
35	Shouhong Zhu	2021	CRISPR, RNAi	*GhAlaRP*	Shortening	[52]
36	Yao Wang	2021	RNAi	*GhKNL1*	Increasing	[53]
37	Lei Zheng	2020	VIGS	*GhGATL15*	Shortening	[54]
38	Jun-Feng Cao	2020	RNAi	*GhTCP4*	Increasing	[55]
39	Huiru Sun	2020	VIGS	*GhPEL76*	Shortening	[56]
40	Zhi-Hao Liu	2019	RNAi	*GhFP1*	Shortening	[57]
41	Hao Feng	2018	RNAi	*GhHUB2*	Shortening	[58]
42	Verandra Kumar	2018	RNAi	*GhHDA5*	Increasing	[59]
43	Li-Xia Qin	2016	RNAi	*GhGalT1*	Increasing	[60]
44	Yuan Cheng	2016	VIGS	*GhCaM7-like*	Shortening	[61]
45	Gao-Jun Liu	2015	VIGS	*GhΔ15FAD/GhPIS/GhPIK*	Shortening	[62]
46	Ying Zhou	2014	RNAi	*Gh14-3*	Shortening	[63]
47	Juan Hao	2012	RNAi	*GbTCP*	Shortening	[64]
48	Li Pu	2008	RNAi	*GhMYB109*	Shortening	[65]

## Data Availability

The original contributions presented in this study are included in the article. Further inquiries can be directed to the corresponding authors.
